# A comprehensive analysis of the faecal microbiome and metabolome of *Strongyloides stercoralis* infected volunteers from a non-endemic area

**DOI:** 10.1038/s41598-018-33937-3

**Published:** 2018-10-23

**Authors:** Timothy P. Jenkins, Fabio Formenti, Cecilia Castro, Chiara Piubelli, Francesca Perandin, Dora Buonfrate, Domenico Otranto, Julian L. Griffin, Lutz Krause, Zeno Bisoffi, Cinzia Cantacessi

**Affiliations:** 1 Department of Veterinary Medicine, University of Cambridge, Cambridge, United Kingdom; 2 Centre for Tropical Diseases, IRCCS Sacro Cuore-Don Calabria Hospital, Negrar, Verona, Italy; 3 Department of Biochemistry, University of Cambridge, Cambridge, United Kingdom; 4 Department of Veterinary Medicine, University of Bari, Valenzano, Italy; 5 The University of Queensland Diamantina Institute, Translational Research Institute, Brisbane, QLD Australia; 6 Department of Diagnostics and Public Health, University of Verona, Verona, Italy

**Keywords:** Statistical methods, Parasite host response

## Abstract

Data from recent studies support the hypothesis that infections by human gastrointestinal (GI) helminths impact, directly and/or indirectly, on the composition of the host gut microbial flora. However, to the best of our knowledge, these studies have been conducted in helminth-endemic areas with multi-helminth infections and/or in volunteers with underlying gut disorders. Therefore, in this study, we explore the impact of natural mono-infections by the human parasite *Strongyloides stercoralis* on the faecal microbiota and metabolic profiles of a cohort of human volunteers from a non-endemic area of northern Italy (*S*+), pre- and post-anthelmintic treatment, and compare the findings with data obtained from a cohort of uninfected controls from the same geographical area (*S−*). Analyses of bacterial 16S rRNA high-throughput sequencing data revealed increased microbial alpha diversity and decreased beta diversity in the faecal microbial profiles of *S*+ subjects compared to *S−*. Furthermore, significant differences in the abundance of several bacterial taxa were observed between samples from *S*+ and *S−* subjects, and between *S*+ samples collected pre- and post-anthelmintic treatment. Faecal metabolite analysis detected marked increases in the abundance of selected amino acids in *S*+ subjects, and of short chain fatty acids in *S−* subjects. Overall, our work adds valuable knowledge to current understanding of parasite-microbiota associations and will assist future mechanistic studies aimed to unravel the causality of these relationships.

## Introduction

The human gastrointestinal (GI) tract is inhabited by a myriad of bacteria, viruses, archaea, fungi, and other unicellular and multicellular microorganisms, that together form the gut micro- and macrobiota^1–4^. Whilst some members of the microbiota can cause severe disease^5^, most resident bacteria exert a number of specialised functions that are beneficial to the human host, including absorption of nutrients, synthesis of essential organic compounds, development of adaptive immunity and protection against pathogens^6–9^. Disturbances of the composition of the gut microbiota (i.e. dysbiosis) have been unequivocally linked to the onset of a range of gut and systemic diseases, such as chronic autoimmune and allergic disorders, obesity, diabetes and, more recently, multiple sclerosis (MS)^10–13^. On the other hand, with a few exceptions, multicellular organisms residing in the GI tract, such as parasitic worms (=helminths) are mostly considered detrimental to human health, as they can subtract nutrients, damage host tissues and release toxic waste products^14–16^. Nonetheless, in the developing world, infections by parasitic helminths have been associated with a low incidence of allergic and autoimmune diseases, as encompassed by the ‘hygiene hypothesis’^17,18^; this observation has led to the ‘curative’ properties of a range of GI helminths being investigated in a range of clinical trials aimed to develop novel therapeutics against selected chronic inflammatory disorders, such as ulcerative colitis^19,20^, Crohn’s disease^21–24^, coeliac disease^25,26^ and MS^27–31^. Whilst preliminary results from a number of such trials are promising, a thorough understanding of the mechanisms that determine the anti-inflammatory properties of these helminths is necessary to assist the development of new effective therapeutics against these disorders. These properties are predominantly attributed to the ability of parasites and/or their excretory/secretory products to modulate host immune responses to facilitate their long-term establishment in the human gut^32–43^; nevertheless, in recent years, the ability of controlled infections by selected GI helminths to ameliorate clinical signs of chronic inflammation has been hypothesized to stem, at least in part, from direct and/or immune-mediated interactions between parasites and the resident microbial flora (reviewed by^44^). This hypothesis is supported by observations from several studies^45–50^ that have reported associations between human infections by GI parasites (under experimental and natural settings) and shifts in the composition of the human gut microbiota towards a ‘healthy’ phenotype, as well as increased levels of metabolites with anti-inflammatory properties^45–50^. However, information reported to date have been derived from cohorts of human volunteers with underlying chronic gut disorders (e.g. coeliac disease^45–48^) or conditions of malnutrition and/or multi-specific helminth infections and/or exposed to multiple re-infections^50–53^, with likely implications on the ‘steady-state’ of the gut flora of these individuals. Whilst complete elimination of these confounding factors is difficult to achieve in human studies, investigations of the impact that infections by single species of GI helminths exert on the composition of the gut flora of individuals with no clinical evidence of concurring co-infections or underlying gut disorders may help disentangle the causality of parasite-microbiota relationships; in turn, this knowledge may assist the design of mechanistic experiments in available animal models of infection and disease (cf.^54–57^), aimed to achieve a better understanding of the therapeutic properties of parasites.

*Strongyloides stercoralis* is a soil transmitted intestinal nematode estimated to infect ~370 million people worldwide, with higher prevalence (ranging from 10% to 60%) recorded across tropical and subtropical regions^58–61^. The life cycle of *S*. *stercoralis* is complex, in that it involves both free-living and parasitic adult stages^58,62^. In particular, the small intestine of the vertebrate hosts (e.g. humans) harbours adult females only, which reproduce *via* parthenogenesis and lay eggs that hatch immediately, releasing first stage rhabditiform larvae (L1s) that are excreted with the host faeces (reviewed by^58,62^). However, L1s can also develop into invasive filariform larvae that are able to re-infect the host without being excreted (i.e. ‘autoinfection’)^62^. Once in the environment, male L1s develop through four larval stages to free-living adults; conversely, female L1s can either develop through to free-living adults (similarly to males) or reach a developmental stage infective to a new susceptible host, i.e. the infective third-stage larva (L3). Importantly, the new generation of female parasites deriving from sexual reproduction of free-living males and females is inevitably parasitic^58,62^. These infective larvae typically infect humans percutaneously and migrate to the small intestine, where the cycle recommences^58,62^. Autoinfection of a susceptible host can occur at a low level for several years, and is often subclinical or asymptomatic^58,62^ although, in immunosuppressed individuals, parasites can spread to all organs and tissues causing (potentially fatal) ‘disseminated strongyloidiasis’.

Chronic infections by *S*. *stercoralis* provide a golden opportunity to evaluate the effect/s of long-term colonisation by parasitic nematodes on the composition of the human gut microbiota. In this study, we explore the impact of natural infections by *S*. *stercoralis* on the faecal microbiota and metabolic profiles of a cohort of elderly volunteers (with no clinical evidence of concurrent pathologies of infectious or non-infectious origin) from northern Italy. This area is non-endemic for parasitic nematodes, but characterised by the presence of sporadic cases of chronic infections by *S*. *stercoralis* in elderly individuals in which the parasite has persisted through several decades *via* autoinfection^63^. We profiled the faecal microbiota of these subjects pre- and post-anthelmintic treatment with ivermectin, and compared the findings with a control cohort of uninfected individuals from the same geographical area.

## Results

### The composition of the faecal microbiota of human volunteers infected by *Strongyloides stercoralis* pre- and post-anthelmintic treatment

Individual faecal samples were collected from 20 elderly volunteers [74 ± 11 years of age (average ± standard deviation)] with confirmed infections by *S*. *stercoralis* (*S*+), as well as from 11 uninfected volunteers (*S−*) of comparable age and from the same geographical areas (Supplementary Fig. S1). Additional faecal samples were collected from 13 (out of 20) *S*+ subjects 6 months post-anthelmintic treatment. In comparative analyses of the human faecal microbiota pre- and post-treatment, samples from the latter 13 subjects are hereafter referred to as *S*+_*pre-treatment*_ and *S*+_*post-treatment*_, respectively. A total of 44 faecal samples were subjected to microbial DNA extraction and high-throughput Illumina sequencing of the bacterial 16S rRNA gene [i.e. *S*+ = 20 (including *S*+_*pre-treatment*_ = 13), *S*+_*post-treatment*_ = 13 and *S*− = 11] whilst a total of 31 samples [i.e. *S*+ = 14 (including *S*+_*pre-treatment*_ = 8), *S*+_*post-treatment*_ = 7 and *S*− = 10] were subjected to metabolite profiling *via* nuclear magnetic resonance (NMR) and gas chromatography/mass spectrometry (GC-MS) (cf. Supplementary Table S1).

**Figure 1:**
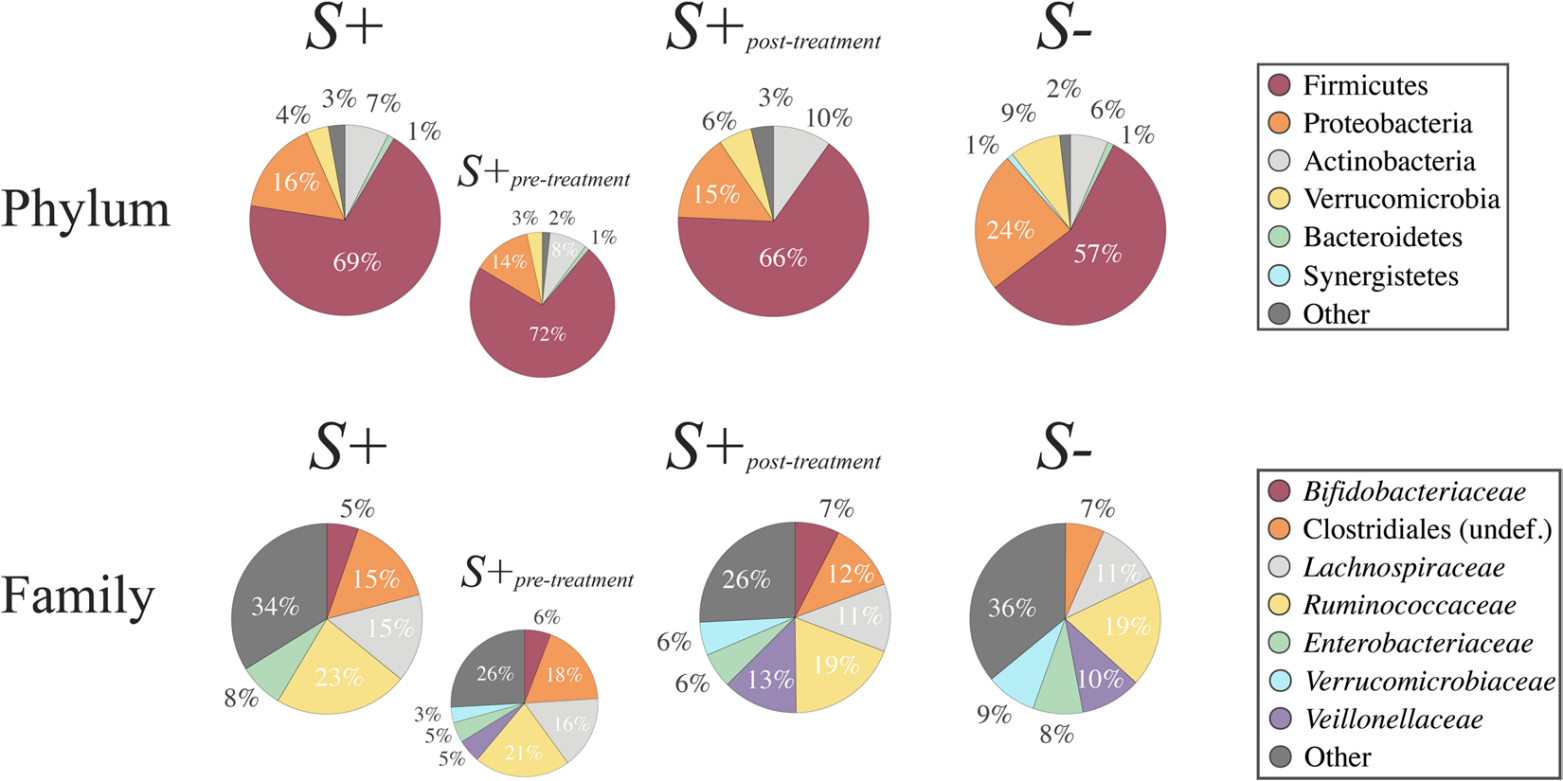
Firmicutes and Proteobacteria are most abundant phyla in the faecal microbiota of all study subjects. Relative abundances of bacterial phyla and families detected in faecal samples from *Strongyloides stercoralis* infected and uninfected subjects (*S*+ and *S*−, respectively) and of the subset of *S*+ subjects that had received anthelmintic treatment (associated sub-circles), both prior to (*S*+_*pre-treatment*_) and 6 months post-ivermectin administration (*S*+_*post-treatment*_). Percentages in individual pie chart sections indicate the relative proportion of a given bacterial phylum or family.

**Figure 2:**
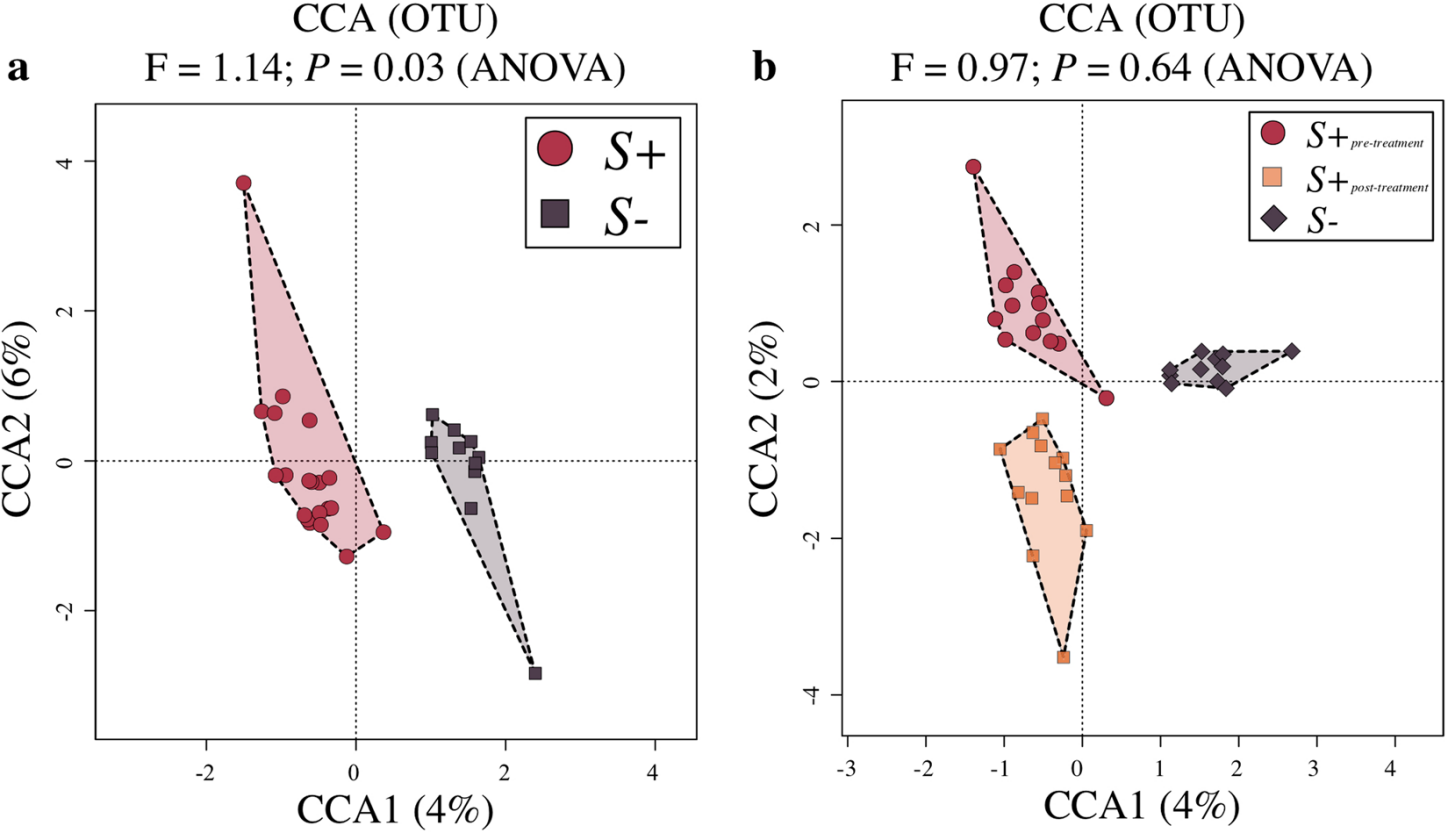
The faecal microbial profiles of *Strongyloides stercoralis*-infected individuals and uninfected controls. Differences between the faecal microbial profiles of *S*. *stercoralis* infected and uninfected subjects (*S*+ and *S*−, respectively) (**a**), and between the microbial profiles of the subset of *S*+ subjects that had received anthelmintic treatment, both prior to (*S*+_*pre-treatment*_) and 6 months post-ivermectin administration (*S*+_*post-treatment*_), and of *S*− subjects, ordinated by supervised Canonical Correspondence Analysis (CCA; **b**).

High-throughput amplicon sequencing yielded a total of 5,717,403 paired-end reads (not shown), of which 1,446,150 high-quality sequences (per sample mean 26,778 ± 13,928) were retained after quality control. Rarefaction curves generated following *in silico* subtraction of low-quality sequences indicated that the majority of faecal bacterial diversity was well represented by the sequence data (Supplementary Fig. S2). These sequences were assigned to 2,630 Operational Taxonomic Units (OTUs) and 15 bacterial phyla, respectively (data available from Mendeley Data at 10.17632/n86dtjvmbv.1). The phyla Firmicutes (64.5% average ± 14.4% standard deviation) and Proteobacteria (18.2 ± 12.8%) were most abundant in all samples analysed, followed by the phyla Actinobacteria (7.9 ± 6.4%), Verrucomicrobia (5.4 ± 7.7%) and Bacteroidetes (1 ± 1.1%) (Fig. 1). At the order level, Clostridiales were most abundant in all samples analysed (54.4 ± 15.2%), and included the two most abundant microbial families, i.e. the *Ruminococcaceae* (20.6 ± 11.6%) and the *Lachnospiraceae* (13.4 ± 8.1%) (Fig. 1). Faecal microbial community profiles were ordinated by Canonical Correspondence Analysis (CCA) (Fig. 2a), that separated samples by infection status (*S*+ and *S*−) (effect size (F) = 1.14, *P* = 0.03). No statistically significant differences between the gut microbial composition of *S*+_*pre-treatment*_, *S*+_*post-treatment*_, and *S*− subjects were detected (F = 0.97, *P* = 0.64; Fig. 2b). Similarly, *S*+_*pre-treatment*_ and *S*+_*post-treatment*_ samples did not show any statistically significant difference in community composition (*P* > 0.05, CCA).

### Infection by *Strongyloides stercoralis* is associated with increased alpha diversity, and decreased beta diversity, of the faecal microbiota

**Figure 3:**
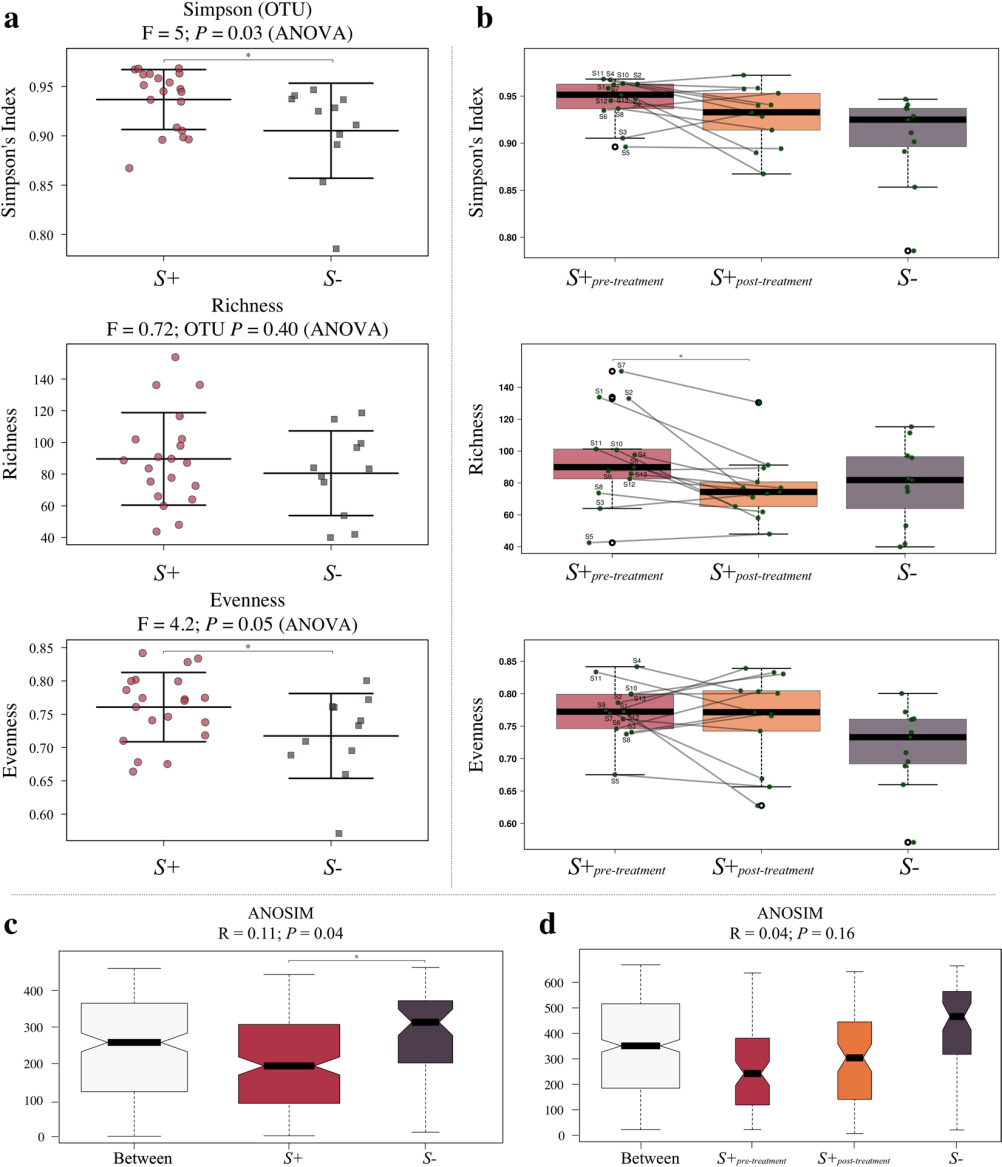
The faecal microbiome of *Strongyloides stercoralis*-infected subjects is characterised by increased alpha diversity and decreased beta diversity, respectively, when compared to that of uninfected controls. (**a**) Differences in overall microbial Simpson (alpha) diversity, and corresponding richness and evenness, between the faecal microbial profiles of *S*. *stercoralis*-infected and uninfected subjects (*S*+ and *S*−, respectively), and (**b**) between the faecal microbial profiles of the subset of *S*+ subjects that had received anthelmintic treatment, both prior to (*S*+_*pre-treatment*_) and 6 months post-ivermectin administration (*S*+_*post-treatment*_), and of *S*− subjects. (**c**) Differences in microbial beta diversity between *S*+ and *S*− samples, as well as (**d**) between samples from *S*+_*pre-treatment*_, *S*+_*post-treatment*_ and *S*−. The bold and black horizontal lines in the boxplots refer to the respective mean (i.e. Simpson’s Index, richness, and evenness) associated with the corresponding group, with top and bottom whiskers representing the standard deviation. Points connected by lines in (**b**) refer to samples collected from the same study participant pre- and post-anthelmintic treatment. Significant differences between study groups are marked by an asterisk (*).

Microbial alpha diversity, measured through Simpson’s index, and evenness were significantly increased in the faecal microbiota of *S*+ volunteers when compared to that of *S*− (F = 5, *P* = 0.03 and F = 4.2, *P* = 0.05 respectively) (Fig. 3a). Faecal microbial richness was not significantly different between *S*+ and *S*− subjects, albeit a trend towards increased richness in samples from *S*+ subjects was observed. Simpson diversity and richness were decreased in samples from *S*+_*post-treatment*_ compared to *S*+_*pre-treatment*_, although only the latter was significant (*P* < 0.001; mixed effect linear regression) (Fig. 3b). Compared to *S*−, *S*+_*post-treatment*_ samples showed a trend towards increased Simpson diversity and evenness, while richness was lower in samples post-treatment when compared to samples from uninfected subjects (Fig. 3b). Microbial beta diversity was significantly lower in samples from *S*+ subjects when compared to *S*− subjects (effect size (R) = 0.11, *P* = 0.04; Fig. 3c), whilst beta diversity in *S*+_*post-treatment*_ samples was higher than that in *S*+_*pre-treatment*_ but lower than that in *S*− samples, albeit not significantly (Fig. 3d).

### *Strongyloides stercoralis* infection is associated with expanded populations of *Leuconostocaceae*, *Ruminococcaceae*, *Paraprevotellaceae* and *Peptococcus* and reduced Pseudomonadales

**Figure 4:**
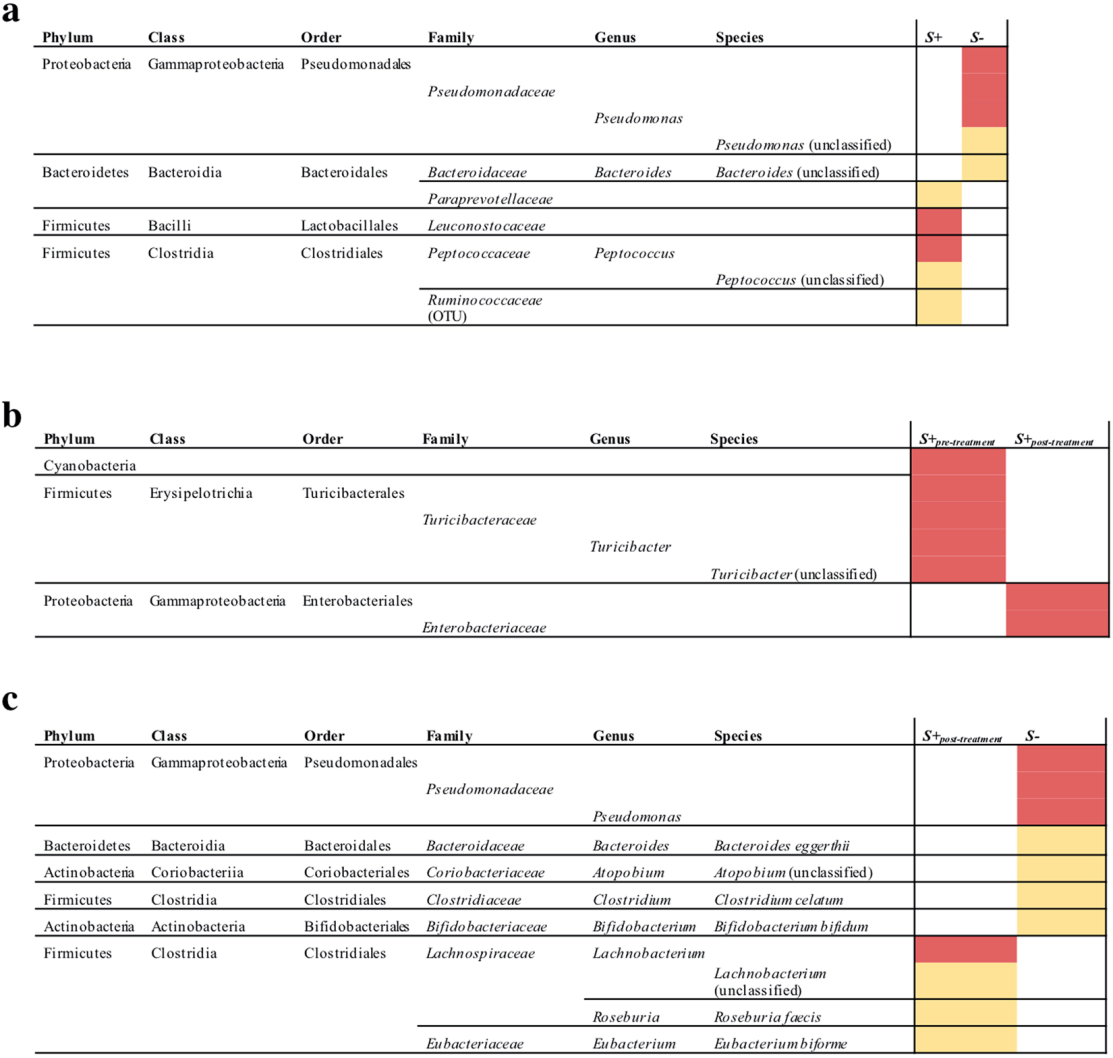
Differentially abundant bacterial taxa in the faecal microbiota of *Strongyloides stercoralis* (**a**) infected and uninfected subjects (*S*+ and *S*−, respectively), (**b**) infected subjects pre- and post anthelmintic treatment with ivermectin (*S*+_*pre-treatment*_ and *S*+_*post-treatment*_, respectively), and (**c**) infected subjects post-anthelmintic treatment and uninfected subjects (*S*+_*post-treatment*_ and *S*− respectively), based on Linear discriminant analysis Effect Size (LEfSe) analysis. Colours correspond to Linear Discriminant Analysis (LDA) scores of 4 or higher (red) and 3.5 to 4 (yellow).

Linear discriminant analysis Effect Size (LEfSe) revealed significant differences in the abundance of individual microbial taxa (phylum to species level) between *S*+ and *S*− subjects (Fig. 4a). In particular, the faecal microbiota of *S*− subjects was significantly enriched for populations of bacteria belonging to the order Pseudomonadales (genus *Pseudomonas*) and an unidentified species belonging to the genus *Bacteroides* (Fig. 4a); conversely, bacteria belonging to the families *Leuconostocaceae*, *Ruminococcaceae*, *Paraprevotellaceae* and to the genus *Peptococcus*, amongst others, were significantly higher in the faecal microbiota of *S*+ subjects (Fig. 4a). In addition, in samples from *S*+_*post-treatment*_ subjects, a significant decrease of bacteria belonging to the order Turicibacterales (genus *Turicibacter*) and an increase of Enterobacteriales (in particular associated to the genus *Shigella*) were observed compared to corresponding samples from *S*+_*pre-treatment*_ (Fig. 4b and Supplementary Fig. S3). Additionally, differences were observed between the microbial profiles of *S*+_*post-treatment*_ samples when compared to *S*−, with the latter displaying increased levels of bacteria belonging to the order Pseudomonadales and genus *Atopobium*, as well as *Bacteroides eggerthii*, *Clostridium celatum* and *Bifidobacterium bifidum*, and decreased levels of *Lachnobacterium*, *Roseburia faecis*, and *Eubacterium biforme*, respectively (Fig. 4c).

### The faecal metabolome of *Strongyloides stercoralis* infected volunteers is characterized by low levels of short chain fatty acids (SCFAs) and increased amino acid abundance

**Figure 5:**
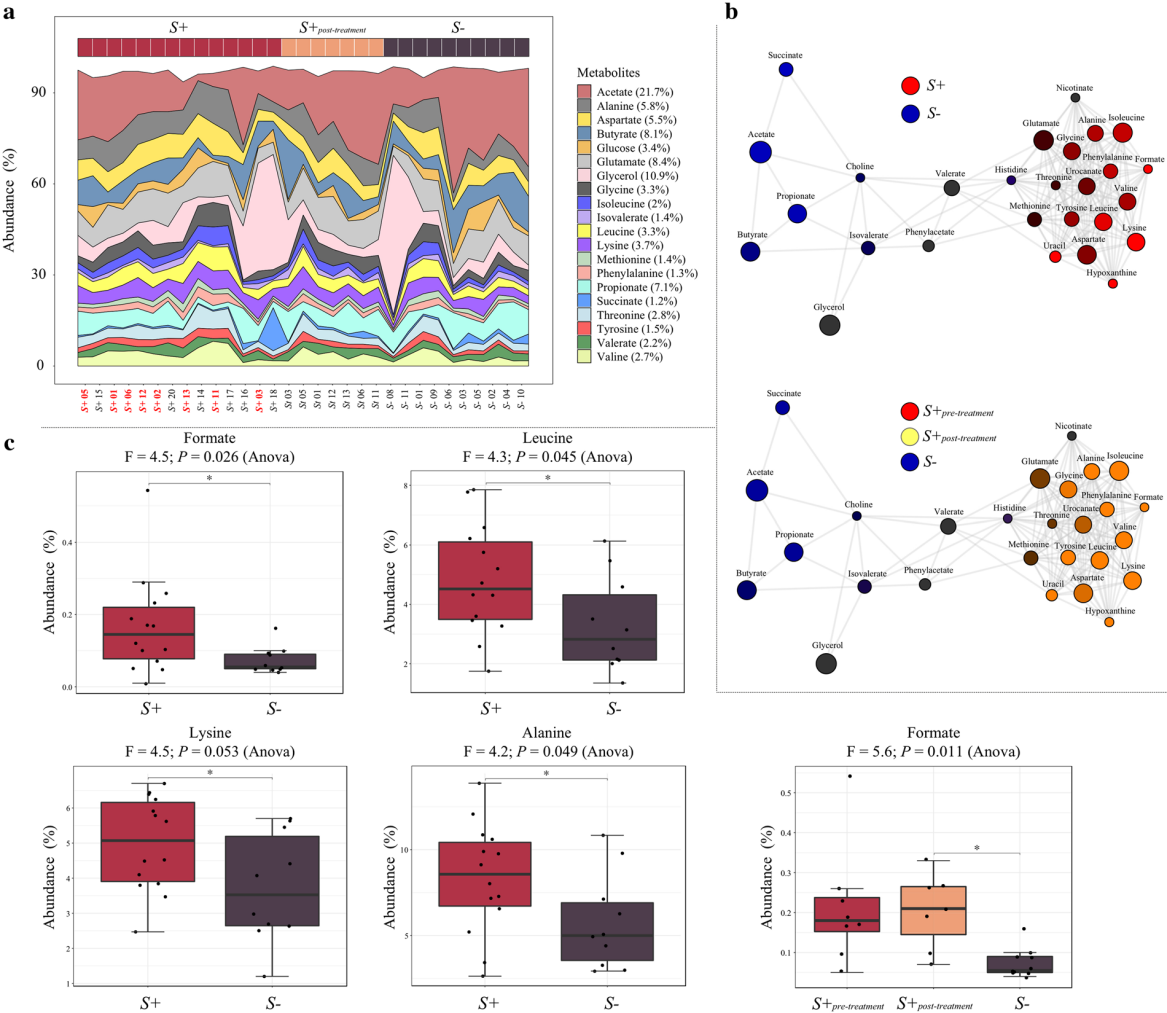
Decreased levels of selected short chain fatty acids (SCFAs) and increased levels of amino acids are associated with *Strongyloides stercoralis* infection. (**a**) Area plot indicating the abundance (expressed as percentage) of metabolites detected by nuclear magnetic resonance analysis (NMR) in faecal samples from *S*. *stercoralis*-infected and uninfected subjects (*S*+ in red, and *S*− in purple), as well as from the subset of *S*+ subjects that had received anthelmintic treatment, both prior to (*S*+_*pre-treatment*,_ red sample label) and 6 months post-ivermectin administration (*S*+_*post-treatment*_, in orange). (**b**) Network analysis displaying associations between individual or clusters of metabolites and sample groups (i.e. *S*+ in red, *S*− in blue [top network], *S*+_*pre-treatment*_ in red, *S*+_*post-*treatment_ in yellow and *S*− in blue [bottom network]). For metabolites associated with multiple sample groups, the respective circle colours are mixed according to the strength of the association. (**c**) Differentially abundant metabolites detected in faecal samples from *S*+ and *S*−, as well as *S*+_*pre-treatment*_, *S*+_*post-treatment*_, and *S*− subjects, determined using ANOVA. The bold and black horizontal lines in the boxplots refer to the mean of percentage abundance of metabolite associated with the corresponding group, with top and bottom whiskers representing the standard deviation. Significant differences between study groups are marked by an asterisk (*).

A total of 28 metabolites were identified by NMR across all samples (Fig. 5a); these were subjected to Principal Coordinates Analysis (PCoA), which unveiled no marked differences in faecal metabolic profiles between samples from *S*+ and *S*− subjects, as well as from *S*+_*pre-treatment*_, *S*+_*post-treatment*_, and *S*− subjects (data not shown). However, association network analysis of *S*+ and *S*− samples indicated clustering of several faecal metabolites according to the infection status of the study subjects. In particular, 13 metabolites (alanine, isoleucine, glycine, phenylalanine, formate, valine, tyrosine, leucine, lysine, uracil, hypoxanthine, and aspartate), were positively correlated with each other and associated with the faecal metabolic profiles of *S*+ subjects (Fig. 5b), whereas four (the SCFAs propionate, butyrate, and acetate, as well as succinate) were also positively correlated with each other, and associated with faecal samples from *S*− subjects (Fig. 5b). When applied to the faecal metabolic profiles of *S*+_*pre-treatment*_, *S*+_*post-treatment*_ and *S*− subjects, network analysis associated the 13 metabolites described above (previously associated with *S*+) with both *S*+_*pre-treatment*_ and *S*+_*post-treatment*_, whereas the SCFAs remained associated with the metabolic profiles of *S*− subjects (Fig. 5b). Analysis of differentially abundant metabolites between *S*+_*pre-treatment*_, *S*+_*post-treatment*_, and *S*− samples via ANOVA further revealed that 10 (out of 12) metabolites associated with samples from *S*+_*post-treatment*_ subjects were less abundant than in *S*+_*pre-treatment*_ samples, but more abundant when compared to *S*− samples, albeit not significantly (Supplementary Fig. S4). Additionally, alanine, formate, lysine, and leucine were significantly more abundant in samples from *S*+ when compared to *S*− (F = 4.2, *P* = 0.05; F = 4.5, *P* = 0.03; F = 4.5, *P* = 0.05; F = 4.3, *P* = 0.05 respectively) whilst formate was significantly more abundant in *S*+_*post-treatment*_ compared to *S*− subjects (F = 5.6, *P* = 0.01); nevertheless, these differences were not significant following p-value correction for multiple testing (FDR > 0.05) (Fig. 5c).

Using GC-MS, a total of 13 fatty acids were detected across all analysed faecal samples, including lauric acid (C12:0), myristic acid (C14:0), pentadecanoic acid (C15:0, C15:0 iso, and C15:0 ante), palmitic acid (C16:0), margaric acid (C17:0, C17:0 iso, C17:0 ante), stearic acid (C18:0), oleic acid (C18:1), linoleic acid (C18:2), and arachidic acid (C20:0) (Supplementary Fig. S5). No significant associations between any of the analysis groups were detected using PCoA and no significant differences in the relative abundance of individual metabolites were observed between samples from *S*+ and *S*− subjects, as well as *S*+_*pre-treatment*_, *S*+_*post-treatment*_, and *S*− subjects (data not shown).

## Discussion

In this study, we aimed to determine the effect/s that chronic, monospecific infections by the parasitic nematode *S*. *stercoralis* exert/s on the faecal microbiome and metabolome of human volunteers from a non-endemic area of Europe, and establish whether such effects are reversed following the administration of anthelmintic treatment. Whilst the overall composition of the faecal microbiota of *Strongyloides*-infected and uninfected volunteers enrolled in this study largely reflected that of human subjects harbouring GI helminths under natural or experimental conditions of infections described in previous investigations, the relatively low proportions of Bacteroidetes observed in our samples contrast findings from some previously published reports^47,48,50,51,53^. However, this discrepancy may be accounted for by differences in mean age of the cohorts enrolled in our (72 years of age) *versus* previous studies (i.e. 11–51 years of age; cf.^47,48,50,51,53^); indeed, a decline in the relative abundance of Bacteroidetes in the gut microbiota of aging subjects has been documented in several studies^64–67^, and is thus considered ‘physiological’ in the age group enrolled in our experiment.

In spite of the overall similarities in the composition of the faecal microbiota of *S*+ and *S*− subjects at phylum level, CCA analysis revealed differences in the microbial profiles of these two groups, thus indicating that *S*. *stercoralis* infection was associated with shifts in the relative abundance of individual gut bacterial species. Indeed, microbial alpha diversity was significantly higher in the faecal microbiota of *S*+ when compared to *S*−. In particular, the level of microbial alpha diversity detected in the latter group largely reflected that recorded in a cohort of healthy, elderly Italians investigated previously^68^. Conversely, microbial beta diversity was significantly decreased in faecal samples from *S*+ subjects when compared to *S*−. Increased levels of microbial alpha diversity have been repeatedly observed in the gut microbiome of human subjects infected by a range of GI helminths (i.e. *Necator americanus*, *Trichuris trichiura*, and *Ascaris* sp.)^47,48,50^. Since alpha diversity measures are frequently used as proxy of microbiome ‘health’ (with high alpha diversity associated with a mature, homogenous, stable and healthy gut microbial environment^69,70^), it has been proposed that the direct or immune-mediated ability of GI helminths to restore gut homeostasis by promoting increases in microbial richness and evenness may represent one mechanism by which parasites exert their therapeutic properties in individuals with chronic inflammatory disorders^48,49,71^.

The difference in microbial alpha and beta diversity observed between *S*+ and *S*− subjects were determined by dissimilarities in the relative abundance of selected bacterial taxa in the faecal microbial profiles of these study groups. In particular, significantly expanded populations of Clostridia and *Leuconostocaceae* could be detected in the faecal microbiota of *S*+ when compared to *S*− subjects. Notably, selected strains of Clostridia strains have been identified as leading players in the maintenance of gut homeostasis, due to their roles in protecting the gut from pathogen colonisation, mediating host immune system development and modulating immunological tolerance^72^. On the other hand, members of the *Leuconostocaceae*, a family of anaerobic lactic acid-producing bacteria, have been demonstrated to stimulate the release of inflammatory Th1 type cytokines IL-12 and IFN-γ by activated antigen-presenting cells in human peripheral blood mononuclear cells, thus promoting the activation of antimicrobial immune responses^73^. However, a significant decrease in the abundance of *Leuconostocaceae* was recorded in the faecal microbiota of humans infected by hookworms, whipworms and ascarids^51^, contrasting findings from this study and highlighting the need for further investigations in this area. In addition, the lactobacilli, another group of lactic acid-producing bacteria that has been positively associated with parasite colonisation in rodent models of helminth infections in several recently published studies^55,74–82^, was not detected amongst the bacterial populations that were expanded in samples from *S*+ subjects. It must be also pointed out that, thus far and to the best of our knowledge, investigations conducted in human volunteers have not reported significant shifts in the abudance of lactobacilli in the gut microbiota of helminth-infected subjects^45,47,48,50–53^. Whilst expanded populations of lactobacilli following helminth colonisation may represent a rodent-specific response to infection, mechanistic studies conducted, for instance, in microbiota-humanised mouse models of helminth infections may assist clarifying this point.

The faecal microbiota of *S*− subjects was enriched with a number of known opportunistic and/or potentially pathogenic bacteria, including *Bacteroides eggerthii* (higher abundances of which have been linked to increased risk for and severity of colitis in mice^83^) and species within the genus *Pseudomonas*. Expanded populations of opportunistic and potentially pathogenic microbes, coupled with an overall increase in bacterial beta diversity, have been described in the microbiome of aging humans^84,85^ and may therefore underpin our observations.

Notably, whilst administration of ivermectin (a macrocyclic lactone) to a sub-group of *S*+ subjects did not result in significant differences in the levels of microbial alpha- and beta diversity post-anthelmintic treatment (likely due to the limited number of volunteers who agreed to provide further faecal samples 6 months post-treatment), a tendency towards decreased alpha diversity and increased beta diversity, respectively, was observed in *S*+_*post-treatment*_ samples when compared to *S*+_*pre-treatment*_. Nevertheless, an increase in pathogenic bacteria, including *Enterobacteriaceae* (linked to the genus *Shigella*) was observed in samples from *S*+_*post-treatment*_ subjects when compared to *S*+_*pre-treatment*_. This change was also accompanied by an increase in the probiotic *Lachnospiraceae* in *S*+_*post-treatment*_ subjects when compared to *S*−^86^, thus suggesting that anthelmintic treatment may have affected the taxonomic composition of the gut microbiota of previously infected subjects. Conversely, a recent study investigating the impact of treatment with albendazole (a benzimidazole compound) on a large cohort of human volunteers from Indonesia infected by ascarids, whipworms and/or hookworms, detected no differences between the faecal microbial composition of these volunteers and that of a placebo-treated cohort^87^. This discrepancy may be attributable to fundamental differences between the two anthelmintics investigated and/or between parasite species assessed, and/or to sample size limitations; nevertheless, future experiments carried out in large cohorts of volunteers and/or in experimental models of *Strongyloides* infection (i.e. rodents infected by *Strongyloides ratti*) may provide further clarification.

In this study, besides determining the qualitative and quantitative composition of the gut microbiota of *Strongyloides*-infected human volunteers, we also carried out, for the first time in helminth-infected individuals, a comprehensive analysis of the faecal metabolome of the same subjects. Indeed, given that perturbations of the gut microbiota homeostasis are known to exert downstream effects on intestinal metabolism^88,89^, we hypothesized that alterations in gut microbial profiles associated to colonisation by *S*. *stercoralis* might be accompanied by changes in the relative abundance of individual metabolites in faecal samples, with potential implications for the overall health of infected individuals. Whilst analysis via NMR and GC-MS revealed no significant differences in the relative abundance of the vast majority of metabolites identified in samples from *S*+ versus *S*−, a number of amino acids (i.e. leucine, lysine, and alanine) were significantly more abundant in the faecal metabolome of infected individuals when compared to the uninfected cohort. Notably, anthelmintic treatment appeared to only affect the metabolites associated with helminth infection, i.e. amino acids, and thus may suggest that the helminth removal affects both the microbiome and the metabolome. An increased abundance of amino acids in the predicted faecal metabolome of helminth infected-subjects had been previously reported, albeit this information had been indirectly inferred from high-throughput metagenomics sequencing data^50,90^. Amino acids play key roles in the maintenance of the gut microbiome homeostasis and metabolism, since they support the growth and survival of bacteria in the GI tract^91^. Simultaneously, the gut microbiome exerts important functions in the metabolism of alimentary and endogenous proteins that are converted into peptides and amino acids^92,93^. In particular, the most prevalent species involved in amino acid fermentation within the human intestine are bacteria belonging to the class Clostridia^94–96^, that were more abundant in the faecal microbiota of *S*+ subjects. Of the amino acids that were significantly more abundant in the faecal metabolome of *S*+ subjects, lysine and leucine participate in biological pathways that are key to the maintenance of the gut homeostasis^97,98^. In addition, the biological functions of lysine and leucine are closely linked^99^, suggesting a possible correlation with the positive association of both these amino acids with the faecal metabolome of *Strongyloides*-infected subjects.

In contrast with the increased quantities of amino acids observed in the faecal metabolome of *S*+ subjects, the SCFAs acetate, propionate, and butyrate were significantly less abundant in this group compared to *S*−. This observation contradicts findings from a previous study in which these SCFAs were increased in faecal samples from human volunteers with coeliac disease and experimentally infected with the human hookworm, *N*. *americanus*^49^. Given the known anti-inflammatory properties of SCFAs, Zaiss *et al*.^49^ had hypothesized that these molecules may play a role in the therapeutic effects of GI helminths in chronic inflammatory disorders. The discrepancy observed between our study and that by Zaiss *et al*.^49^ may be attributable to differences between the cohorts of human volunteers investigated (acutely vs. chronically infected; middle aged vs. aged), species of parasite under consideration and infection burden (known vs. unknown). In addition, both studies are characterised limited sample sizes that may have contributed to these contrasting results.

In summary, in our study, monospecific, chronic *S*. *stercoralis* infections were associated with global shifts in the composition of the human faecal microbiota, as well as subtle changes in the faecal metabolic profiles of these individuals when compared with those of uninfected control subjects. In addition, anthelmintic treatment resulted in minor alterations of the faecal microbiota and metabolome of these volunteers 6 months post-administration, albeit sample size limitations prevent us from speculating on the effect/s of worm removal on the gut microbiota and metabolome. Future studies with longer monitoring of qualitative and qualitative fluctuations in faecal microbiota post-treatment may assist shedding light on this point. Whilst our findings add valuable knowledge to the emerging area of host-parasite-microbiota interactions, mechanistic studies in experimental models of infection and disease are necessary to shed light on the likely contribution of parasite-associated modifications in gut microbiome and metabolism to the anti-inflammatory properties of parasitic helminths.

## Methods

### Ethics statement

This study was conducted according to the Declaration of Helsinki, and the protocol was reviewed and approved by the Institutional Ethical Review Committee for clinical experimentation for the Province of Verona (Comitato Etico per la Sperimentazione Clinica delle Province di Verona e Rovigo, protocol number 34678). Written informed consent was obtained from all subjects enrolled in the study.

### Study area and characteristics of the population

Individual faecal samples from 20 volunteers (from 4 regions in northern Italy) with confirmed infections by *S*. *stercoralis* (*S*+) as assessed by Real-Time PCR (rtPCR; cf.^100^), performed at the Centre for Tropical Diseases of the Sacro Cuore Hospital (Negrar, Italy) during routine screening, were examined for microbiota and metabolite profiling as described below. Of these volunteers, 15 were from the Veneto region, three from Lombardia, one from Piemonte, and one from Emilia-Romagna (Supplementary Fig. S1). Subjects were both men (*n* = 12) and women (*n* = 8) of an average age of 74 (range 49–86 ± 11.5) (Supplementary Fig. S1) with no overt symptoms of GI disease and no recent history of anthelmintic treatment. Briefly, immediately following collection of individual faecal samples from each of these volunteers, aliquots (~250 mg) were examined for evidence of patent infections by GI helminths (*S*. *stercoralis*, *Strongyloides fuelleborni*, *N*. *americanus*, *Ancylostoma duodenale*, *Trichostrongylus* spp., *Ternidens deminutus*, and *Oesophagostomum* spp.) using the Agar Plate Copro-Culture Method (http://www.tropicalmed.eu), whilst rtPCR analyses were conducted to detect possible co-infections with *Schistosoma* spp. and *Hymenolepis nana*^100,101^. The remainders of each sample were stored at −80 °C for subsequent microbiota and metabolite profiling (see below). Patent infections by *S*. *stercoralis* were unequivocally confirmed by DNA extractions from individual faecal samples (see below) followed by rtPCR targeting the 18S rRNA gene^100^. Upon confirmation of diagnosis, infected volunteers were treated with ivermectin (Stromectol®, Merck Sharp & Dohme BV, The Netherlands). From 13 (out of 20) *S*+ subjects (9 men and 4 women; average age of 76, range 60–84 ± 8.4, referred to as *S*+_*pre-treatment*_) further individual samples were collected 6 months post-treatment (referred to as *S*+_*post-treatment*_) (Supplementary Fig. S1) and processed as described above. Samples that were negative for patent *S*. *stercoralis* infection at this time were progressed to microbiota and metabolite profiling (see below). In addition, individual faecal samples from 11 uninfected volunteers (*S*−) from the Veneto region (5 men and 6 women; average age of 65, range 53–86 ± 10.7; Supplementary Fig. S1) were included for comparative analyses. These volunteers had no overt symptoms of GI disease or any other concomitant disease and had no recent history of antibiotic treatment.

### DNA extractions and bacterial 16S rRNA gene Illumina sequencing

Genomic DNA was extracted directly from 200 mg of each faecal sample using the MagnaPure LC.2 instrument (Roche Diagnostic, Monza, Italy), following the manufacturer’s instructions, and the DNA isolation kit I (Roche) and stored at −80 °C until further processing. High-throughput sequencing of the V3-V4 hypervariable region of the bacterial 16S rRNA gene was performed by Eurofins Genomics on an Illumina MiSeq platform according to the standard protocols with minor adjustments. Briefly, the V3-V4 region was PCR-amplified using universal primers^102^, that contained the adapter overhang nucleotide sequences for forward (TACGGGAGGCAGCAG) and reverse primers (CCAGGGTATCTAATCC). Amplicons were purified using AMPure XP beads (Beckman Coulter) and set up for the index PCR with Nextera XT index primers (Illumina). The indexed samples were purified using AMPure XP beads (Beckman Coulter) and quantified using the Fragment Analyzer Standard Sensitivity NGS Fragment Analysis Kit (Advanced Analytical) and equal quantities from each sample were pooled. The resulting pooled library was quantified using the Agilent DNA 7500 Kit (Agilent), and sequenced using the v3 chemistry (2 × 300 bp paired-end reads, Illumina).

### Bioinformatics and statistical analyses

Raw paired-end Illumina reads were trimmed for 16S rRNA gene primer sequences using Cutadapt (https://cutadapt.readthedocs.org/en/stable/) and sequence data were processed using the Quantitative Insights Into Microbial Ecology 2 (QIIME2-2018.4; https://qiime2.org) software suite^103^. Successfully joined sequences were quality filtered, dereplicated, chimeras identified, and paired-end reads merged in QIIME2 using DADA2^104^. Sequences were clustered into OTUs on the basis of similarity to known bacterial sequences available in the Greengenes database (v13.8; http://greengenes.secondgenome.com/; 99% sequence similarity cut-off); sequences that could not be matched to references in the Greengenes database were clustered *de novo* based on pair-wise sequence identity (99% sequence similarity cut-off). The first selected cluster seed was considered as the representative sequence of each OTU. The OTU table with the assigned taxonomy was exported from QIIME2 alongside a weighted UniFrac distance matrix. Singleton OTUs were removed prior to downstream analyses. Cumulative-sum scaling (CSS) was applied, followed by log2 transformation to account for the non-normal distribution of taxonomic counts data. Statistical analyses were executed using the Calypso software^105^ (cgenome.net/calypso/); samples were investigated using the taxonomic visualisation tool KRONA^106^ ordinated in explanatory matrices using supervised CCA including infection/treatment status as explanatory variables. Differences in bacterial alpha diversity (Simpson’s index) between study groups (*S*+ and *S*−, as well as *S*+_*pre-treatment*_, corresponding *S*+_*post-treatment*_, and *S*−) were evaluated based on rarefied data (read depth of 6063) and using analysis of variance (ANOVA); F-Tests were used to statistically assess the equality of assessed means (i.e. effect size). To take into account the paired nature of samples from *S*+_*pre-treatment*_ and *S*+_*post-treatment*_, differences between these sets were assessed using linear mixed effect regression. Differences in beta diversity (weighted UniFrac distances) were identified using Analysis of Similarity (ANOSIM) and effect size indicated by an R-value (between −1 and + l, with a value of 0 representing the null hypothesis^107^). Differences in the abundance of individual microbial taxa between groups were assessed using the LEfSe workflow^108^, taking into account the paired nature of *S*+_*pre-treatment*_ and *S*+_*post-treatment*_ samples.

### Metabolite extraction

Metabolites were extracted from 200 mg aliquots of each faecal sample using a methanol–chloroform–water (2:2:1) procedure. 600 μl of methanol–chloroform mix (2:1 v:v) were added, samples were homogenised using stainless steel beads and sonicated for 15 min at room temperature. 200 μl each of chloroform and water were added, the samples were centrifuged and the separated aqueous and lipid phases were collected. The procedure was repeated twice, and the aqueous and lipid fractions from each extraction were pooled. The aqueous layer was dried in a vacuum concentrator (Concentrator Plus, Eppendorf), while the lipid fraction was left to dry overnight at room temperature.

### Nuclear Magnetic Resonance analysis of aqueous extracts

The dried aqueous fractions were re-dissolved in 600 μl D_2_O, containing 0.2 mM sodium-3-(tri-methylsilyl)−2,2,3,3-tetradeuteriopropionate (TSP) (Cambridge Isotope Laboratories, MA, USA) as an internal standard and phosphate buffer (40 mM NaH_2_PO_4_/160 mM Na_2_HPO_4_). The samples were analysed using an AVANCE II+NMR spectrometer operating at 500.13 MHz for the ^1^H frequency and 125.721 MHz for the ^13^C frequency (Bruker, Germany) using a 5 mm TXI probe. The instrument is equipped with TopSpin 3.2. Spectra were collected using a solvent suppression pulse sequence based on a one-dimensional nuclear Overhauser effect spectroscopy (NOESY) pulse sequence to saturate the residual 1 H water signal (relaxation delay = 2 s, t1 increment = 3 us, mixing time = 150 ms, solvent pre-saturation applied during the relaxation time and the mixing time). One hundred and twenty-eight transients were collected into 16 K data points over a spectral width of 12 ppm at 27 °C. In addition, representative samples of each data set were also examined by two-dimensional Correlation Spectroscopy (COSY), using a standard pulse sequence (cosygpprqf) and 0.5 s water presaturation during relaxation delay, 8 kHz spectral width, 2048 data points, 32 scans per increment, 512 increments. Peaks were assigned using the COSY spectra in conjunction with reference to previous literature and databases and the Chenomx spectral database contained in Chenomx NMR Suite 7.7 (Chenomx, Alberta, Canada). 1D-NMR spectra were processed using TopSpin. Free induction decays were Fourier transformed following multiplication by a line broadening of 1 Hz, and referenced to TSP at 0.0 ppm. Spectra were phased and baseline corrected manually. The integrals of the different metabolites were obtained using Chenomx. Metabolites were normalised to total area and differential abundance of metabolites between *S*+ and *S*− subjects, as well as *S*+_*post-treatment*_ and *S*− subjects identified using ANOVA. F-Tests were used to statistically assess the equality of assessed means, while differences between *S*+_*pre-treatment*_ and *S*+_*post-treatment*_ were determined through paired t-test to account for the paired nature of these samples. Associations among metabolites in the faecal metabolome of each sample group were identified by prediction of correlation networks in Calypso^105^ (cgenome.net/calypso/). In particular, networks were constructed to identify clusters of co-occurring metabolites based on their association with infection status (i.e., samples from *S*+ and *S*−, as well as *S*+_*pre-treatment*_, *S*+_*post-treatment*_ and *S*− subjects). Metabolites and explanatory variables were represented as nodes, relative abundance as node size, and edges represented positive associations, while nodes were coloured according to infection status. Metabolite abundances were associated with infection status using Pearson’s correlation. Nodes were then coloured based on the strength of the association (i.e. Spearman’s rho correlation) with infection status. Networks were generated by computing associations between taxa using Spearman’s rho and the resulting pairwise correlations were converted into dissimilarities and then used to ordinate nodes in a two-dimensional plot by PCoA. Therefore, correlating nodes were located in close proximity and anti-correlating nodes were placed at distant locations in the network.

### Gas Chromatography–Mass Spectrometry analysis of organic extracts

100 μl of D-25 tridecanoic acid (200 μM in chloroform), 650 μl of chloroform/methanol (1:1 v/v) and 125 μl BF_3_/methanol (Sigma-Aldrich) were added to 100 μl organic extract dissolved in chloroform/methanol (1:1 v/v) (half of the organic material extracted for each sample). The samples were then incubated at 80 °C for 90 min. 500 μl H_2_O and 1 ml hexane were added and each vial mixed and the two phases separated. The organic layer was evaporated to dryness before reconstitution in 200 μl hexane for analysis. Using a Trace GC Ultra coupled to a Trace DSQ II mass spectrometer (Thermo Scientific, Hemel Hempstead, UK), 2 μl of the derivatised organic metabolites were injected onto a TR-fatty acid methyl ester (FAME) stationary phase column (Thermo Electron; 30 m × 0.25 mm ID × 0.25 μm; 70% cyanopropyl polysilphenylene-siloxane) with a split ratio of 20. The injector temperature was 230 °C and the helium carrier gas flow rate was 1.2 ml/min. The column temperature was 60 °C for 2 min, increased by 15 °C/min to 150 °C, and then increased at a rate of 4 °C/min to 230 °C (transfer line = 240 °C; ion source = 250 °C, EI = 70 eV). The detector was turned on after 240 s, and full-scan spectra were collected using 3 scans/s over a range of 50–650 *m*/*z*. Peaks were assigned using Food Industry FAME Mix (Restek 6098). GC–MS chromatograms were analysed using Xcalibur, version 2.0 (Thermo Fisher), integrating each peak individually, and normalised to total area. The set of metabolic profiles obtained were analysed by univariate analysis. Differential abundance of metabolites between analysis groups was identified using ANOVA, and F-Tests were used to statistically assess the equality of assessed means. Associations among metabolites identified in the faecal metabolome of each sample group were identified by prediction of correlation networks in Calypso^105^ (cgenome.net/calypso/).

## Electronic supplementary material

Supplementary Files
