# Author Correction: A comprehensive analysis of the faecal microbiome and metabolome of *Strongyloides stercoralis* infected volunteers from a non-endemic area

**DOI:** 10.1038/s41598-019-43508-9

**Published:** 2019-06-07

**Authors:** Timothy P. Jenkins, Fabio Formenti, Cecilia Castro, Chiara Piubelli, Francesca Perandin, Dora Buonfrate, Domenico Otranto, Julian L. Griffin, Lutz Krause, Zeno Bisoffi, Cinzia Cantacessi

**Affiliations:** 1 Department of Veterinary Medicine, University of Cambridge, Cambridge, United Kingdom; 2 Centre for Tropical Diseases, IRCCS Sacro Cuore-Don Calabria Hospital, Negrar, Verona Italy; 3 Department of Biochemistry, University of Cambridge, Cambridge, United Kingdom; 4 Department of Veterinary Medicine, University of Bari, Valenzano, Italy; 5 The University of Queensland Diamantina Institute, Translational Research Institute, Brisbane, QLD Australia; 6 Department of Diagnostics and Public Health, University of Verona, Verona, Italy

Correction to: *Scientific Reports* 10.1038/s41598-018-33937-3, published online 23 October 2018

The original version of this Article contained an error in Affiliation 2, which was incorrectly given as ‘Centre for Tropical Diseases, Sacro Cuore-Don Calabria Hospital, Negrar, Verona, Italy’. The correct affiliation is listed below:

Centre for Tropical Diseases, IRCCS Sacro Cuore-Don Calabria Hospital, Negrar, Verona, Italy.

This error has now been corrected in the HTML and PDF versions of the Article, and in the accompanying Supplementary Files.

