# Supplementary material for: A comprehensive analysis of the faecal microbiome and metabolome of Strongyloides stercoralis infected volunteers from a non-endemic area
Source: Sci Rep. 2018 Oct 23;8:15651. doi: 10.1038/s41598-018-33937-3 (PMC6199319; doi:10.1038/s41598-018-33937-3)
Supplement: Supplementary file 1 — Supplementary Files [file 41598_2018_33937_MOESM1_ESM.docx]

**A comprehensive analysis of the faecal microbiome and metabolome of *Strongyloides stercoralis* infected volunteers from a non-endemic area**

**Supplementary information**

**Timothy P. Jenkins^1^, Fabio Formenti^2^, Cecilia Castro^3^, Chiara Piubelli^2^, Francesca Perandin^2^, Dora Buonfrate^2^, Domenico Otranto^4^, Julian L. Griffin^3^, Lutz Krause^5^, Zeno Bisoffi^2,6^, Cinzia Cantacessi^1^**

^1^Department of Veterinary Medicine, University of Cambridge, Cambridge, United Kingdom; ^2^Centre for Tropical Diseases, IRCCS Sacro Cuore-Don Calabria Hospital, Negrar, Verona, Italy; ^3^Department of Biochemistry, University of Cambridge, Cambridge, United Kingdom; ^4^Department of Veterinary Medicine, University of Bari, Valenzano, Italy; ^5^The University of Queensland Diamantina Institute, Translational Research Institute, Brisbane, QLD, Australia; ^6^Department of Diagnostics and Public Health, University of Verona, Verona, Italy

Correspondence and requests for materials should be addressed to C.C. ([cc779@cam.ac.uk](mailto:cc779@cam.ac.uk))

**Supplementary Table S1: | Metadata associated with the samples analysed in this study.** Available metadata associated with faecal samples from *Strongyloides stercoralis*-infected and uninfected subjects (*S+* and *S*-, respectively), as well as from the subset of *S*+ subjects that had received anthelmintic treatment, both prior to (*S+_pre-treatment_*; in red) and 6 months post-ivermectin administration (*S+_post-treatment_*).

| **Subject** | **Infection status** | **Treatment status** | **City** | **Region** | **Age** | **Sex** |
| --- | --- | --- | --- | --- | --- | --- |
| ***S*+*pre-treatment*01*** | Positive | Untreated | Brescia | Lombardia | 82 | Male |
| ***S*+*pre-treatment*02*** | Positive | Untreated | Verona | Veneto | 81 | Male |
| ***S*+*pre-treatment*03*** | Positive | Untreated | Novara | Piemonte | 67 | Male |
| ***S*+*pre-treatment*04** | Positive | Untreated | Verona | Veneto | 83 | Male |
| ***S*+*pre-treatment*05*** | Positive | Untreated | Bologna | Emilia | 60 | Female |
| ***S*+*pre-treatment*06*** | Positive | Untreated | Brescia | Lombardia | 84 | Female |
| ***S*+*pre-treatment*07** | Positive | Untreated | Verona | Veneto | 75 | Male |
| ***S*+*pre-treatment*08** | Positive | Untreated | Verona | Veneto | 81 | Male |
| ***S*+*pre-treatment*09** | Positive | Untreated | Verona | Veneto | 69 | Male |
| ***S*+*pre-treatment*10** | Positive | Untreated | Verona | Veneto | 84 | Male |
| ***S*+*pre-treatment*11*** | Positive | Untreated | Verona | Veneto | 80 | Male |
| ***S*+*pre-treatment*12*** | Positive | Untreated | Brescia | Lombardia | 80 | Female |
| ***S*+*pre-treatment*13*** | Positive | Untreated | Padova | Veneto | 60 | Female |
| *S*+14* | Positive | Untreated | Verona | Veneto | 86 | Male |
| *S*+15* | Positive | Untreated | Verona | Veneto | 69 | Male |
| *S*+16* | Positive | Untreated | Verona | Veneto | 81 | Male |
| *S*+17* | Positive | Untreated | Verona | Veneto | 59 | Female |
| *S*+18* | Positive | Untreated | Verona | Veneto | 49 | Female |
| *S*+19 | Positive | Untreated | Verona | Veneto | 86 | Female |
| *S*+20* | Positive | Untreated | Verona | Veneto | 63 | Female |
| *S*-01* | Negative | Untreated | Verona | Veneto | 78 | Male |
| *S*-02* | Negative | Untreated | Verona | Veneto | 53 | Female |
| *S*-03* | Negative | Untreated | Verona | Veneto | 58 | Female |
| *S*-04* | Negative | Untreated | Verona | Veneto | 86 | Female |
| *S*-05* | Negative | Untreated | Verona | Veneto | 58 | Female |
| *S*-06* | Negative | Untreated | Verona | Veneto | 68 | Male |
| *S*-07 | Negative | Untreated | Verona | Veneto | 74 | Male |
| *S*-08* | Negative | Untreated | Verona | Veneto | 53 | Female |
| *S*-09* | Negative | Untreated | Verona | Veneto | 58 | Female |
| *S*-10* | Negative | Untreated | Verona | Veneto | 73 | Male |
| *S*-11* | Negative | Untreated | Verona | Veneto | 58 | Male |
| *S*+*post-treatment*01* | Negative | Treated | Brescia | Lombardia | 82 | Male |
| *S*+*post-treatment*02 | Negative | Treated | Verona | Veneto | 81 | Male |
| *S*+*post-treatment*03* | Negative | Treated | Novara | Piemonte | 68 | Male |
| *S*+*post-treatment*04 | Negative | Treated | Verona | Veneto | 83 | Male |
| *S*+*post-treatment*05* | Negative | Treated | Bologna | Emilia | 60 | Female |
| *S*+*post-treatment*06* | Negative | Treated | Brescia | Lombardia | 84 | Female |
| *S*+*post-treatment*07 | Negative | Treated | Verona | Veneto | 75 | Male |
| *S*+*post-treatment*08 | Negative | Treated | Verona | Veneto | 81 | Male |
| *S*+*post-treatment*09 | Negative | Treated | Verona | Veneto | 69 | Male |
| *S*+*post-treatment*10 | Negative | Treated | Verona | Veneto | 84 | Male |
| *S*+*post-treatment*11* | Negative | Treated | Verona | Veneto | 80 | Male |
| *S*+*post-treatment*12* | Negative | Treated | Brescia | Lombardia | 80 | Female |
| *S*+*post-treatment*13* | Negative | Treated | Verona | Veneto | 60 | Female |

| *S*+ | Samples from individuals infected with *Strongyloides stercoralis* |
| --- | --- |
| ***S*+*pre-treatment*** | Samples from individuals infected with *Strongyloides stercoralis* for which samples post-anthelmitic treatment were also made available (i.e. S+*pre-treatment*) |
| *S*+*post-treatment* | Samples from individuals infected with *Strongyloides stercoralis* 6 months post-anthelmintic treatment |
| *S*- | Samples from uninfected individuals within the same geographical region and age group |
| * | Samples included in metabolomic analyses *via* nuclear magnetic resonance and gas chromatography-mass spectrometry |

| **Age** | Mean | Minimum | Maximum | Standard deviation |
| --- | --- | --- | --- | --- |
| *S*+ | 74.0 | 49 | 86 | 10.8 |
| *S*+*pre-treatment* | 75.8 | 60 | 84 | 8.5 |
| *S*+*post-treatment* | 75.9 | 60 | 84 | 8.4 |
| *S*- | 65.2 | 53 | 86 | 10.6 |
| **Overall** | 72.3 | 49 | 86 | 10.9 |

| **Samples subjected to high-throughput bacterial 16S rRNA sequencing** | | | | |  |
| --- | --- | --- | --- | --- | --- |
| Sex | *S*+ | *S*+*pre-treatment* | *S*+*post-treatment* | *S*- | All |
| Male | 12 | 9 | 9 | 5 | 26 |
| Female | 8 | 4 | 4 | 6 | 18 |
| **Overall** | 20 | 13 | 13 | 11 | **44** |
|  |  |  |  |  |  |
| Region | Veneto | Lombardia | Piemonte | Emilia | All |
| *S*+ | 15 | 3 | 1 | 1 | 20 |
| *S*+*pre-treatment* | 8 | 3 | 1 | 1 | 13 |
| *S*+*post-treatment* | 8 | 3 | 1 | 1 | 13 |
| *S*- | 11 | 0 | 0 | 0 | 11 |
| **Overall** | 34 | 6 | 2 | 2 | **44** |

| **Samples subjected to metabolomic analyses** | | | |  |  |
| --- | --- | --- | --- | --- | --- |
| Sex | *S*+ | *S*+*pre-treatment* | *S*+*post-treatment* | *S*- | All |
| Male | 7 | 4 | 3 | 4 | 14 |
| Female | 7 | 4 | 4 | 6 | 17 |
| **Overall** | 14 | 8 | 7 | 10 | **31** |
|  |  |  |  |  |  |
| Region | Veneto | Lombardia | Piemonte | Emilia | All |
| *S*+ | 9 | 3 | 1 | 1 | 14 |
| *S*+*pre-treatment* | 3 | 3 | 1 | 1 | 8 |
| *S*+*post-treatment* | 2 | 3 | 1 | 1 | 7 |
| *S*- | 10 | 0 | 0 | 0 | 10 |
| **Overall** | 21 | 6 | 2 | 2 | **31** |

**Supplementary Fig. S1: | Overview of the study area and characteristics of the population.** Faecal samples were collected from *Strongyloides stercoralis* infected and uninfected subjects (*S+* and *S*-, respectively) and of the subset of *S*+ subjects that had received anthelmintic treatment and were re-sampled 6-months post-ivermectin administration (*S+_post-treatment_*).

**
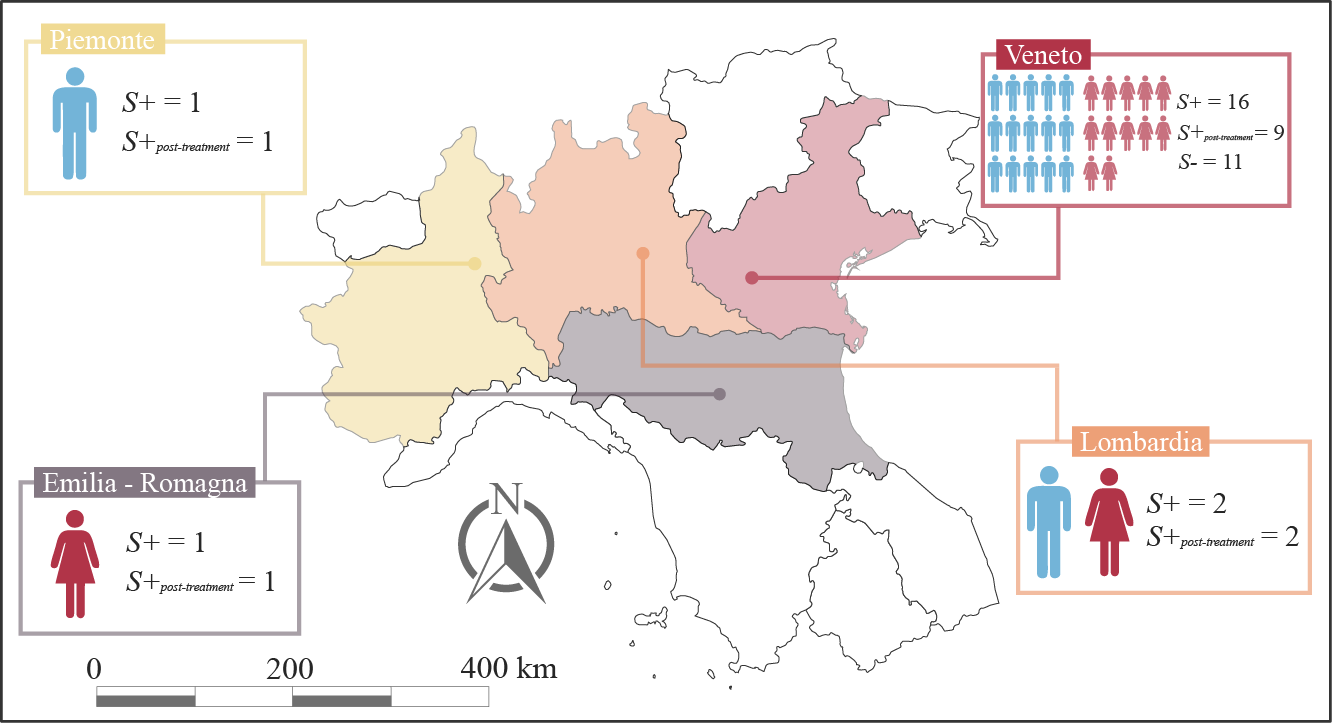
**


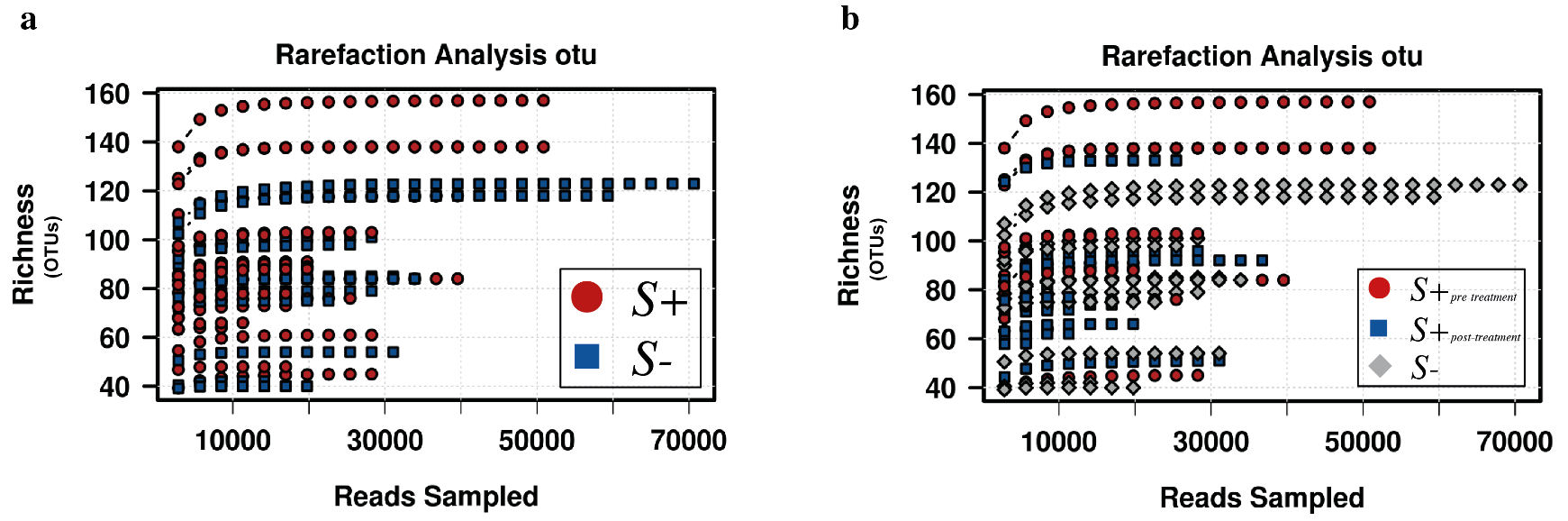
**Supplementary Fig. S2: | Sequence rarefaction analysis.** Rarefaction curves for microbial communities in faecal samples from *Strongyloides stercoralis*-infected and uninfected subjects (*S+* and *S*-, respectively; a), as well as from the subset of *S*+ subjects that had received anthelmintic treatment, both prior to (*S+_pre-treatment_*) and 6 months post-ivermectin administration (*S+_post-treatment_* ;b).

**
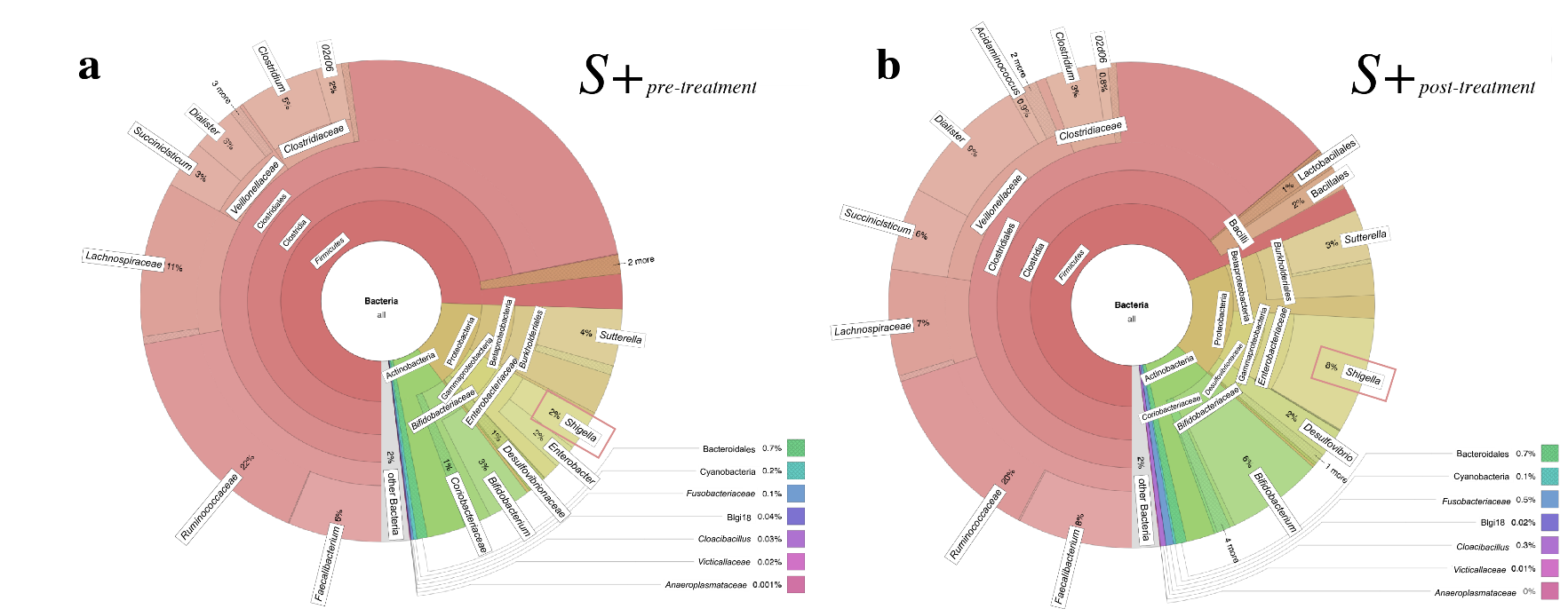
Supplementary Fig. S3: | *Enterobacteriaceae* increase in subjects post-anthelmintic treatment is associated with expanded *Shigella* populations.** KRONA plot indicating taxonomic distribution of taxa associated with samples from subjects infected with *Strongyloides stercoralis* prior to (*S+_pre-treatment_*) and 6 months post-ivermectin administration (*S+_post-treatment_*). The pathogenic bacterial genus *Shigella* is highlighted by a red box.

**Supplementary Fig. S4: | Metabolomic analysis.** Boxplot representation of differentially abundant metabolites detected in faecal samples from subjects infected with *Strongyloides stercoralis* prior to (*S+_pre-treatment_*) and 6 months post-ivermectin administration (*S+_post-treatment_*), as well as uninfected controls. The bold and black horizontal lines in the boxplots refer to the mean of percentage abundance of metabolite associated with the corresponding group, with top and bottom whiskers representing the standard deviation.
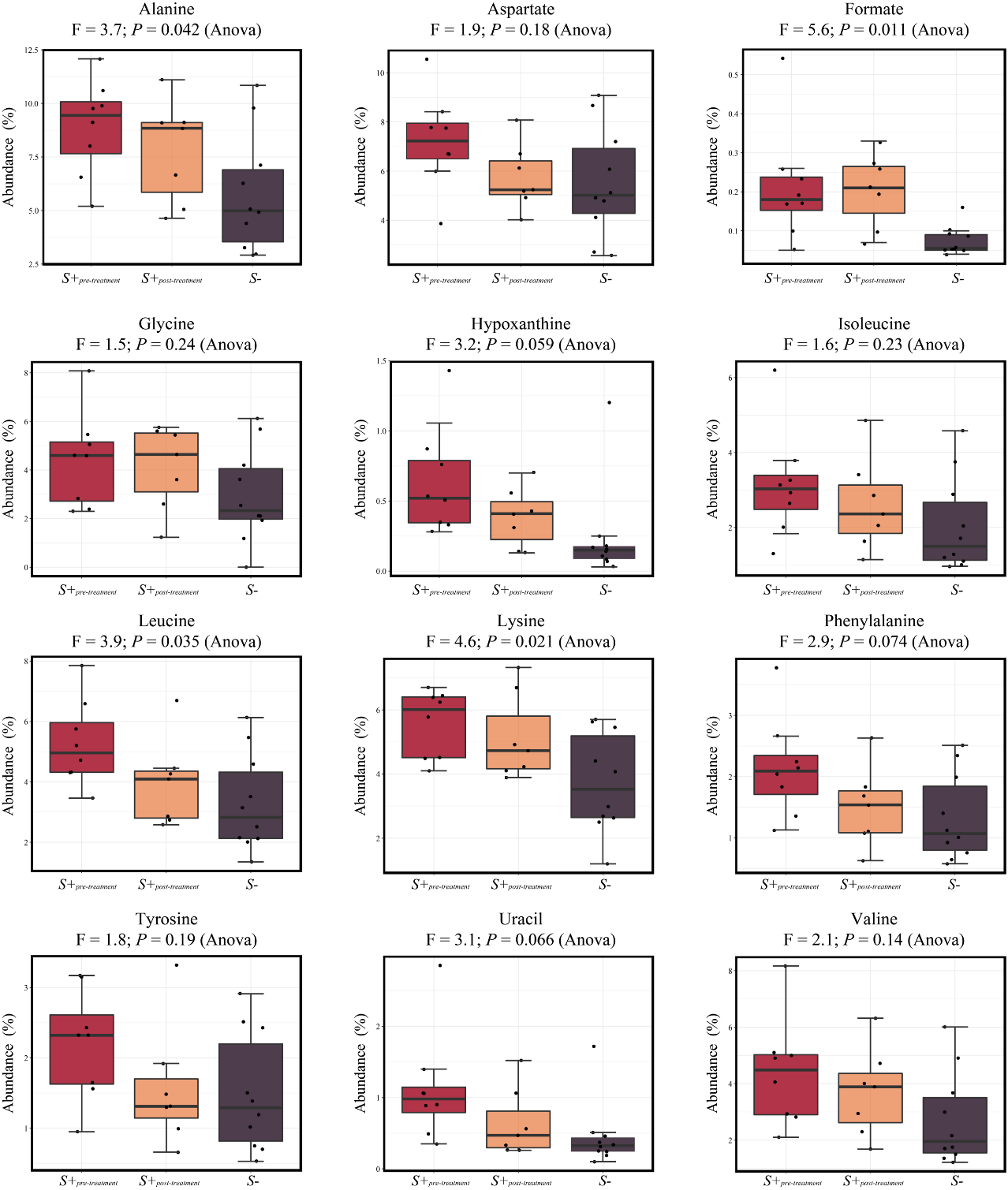


**Supplementary Fig. S5: | Stearic acid and palmitic acid are the most abundant short chain fatty acids in faecal samples from all study subjects.** Area plot indicating the abundance (expressed as percentage) of metabolites detected by nuclear magnetic resonance analysis (NMR) in faecal samples from *S. stercoralis*-infected and uninfected subjects (*S+* in red, and *S*- in purple), as well as from the subset of *S*+ subjects that had received anthelmintic treatment, both prior to (*S+_pre-treatment_*_,_ sample label in red) and 6 months post-ivermectin administration (*S+_post-treatment_*, in orange). Colours within the area plot refer to the respective metabolites, defined in the legend.

**
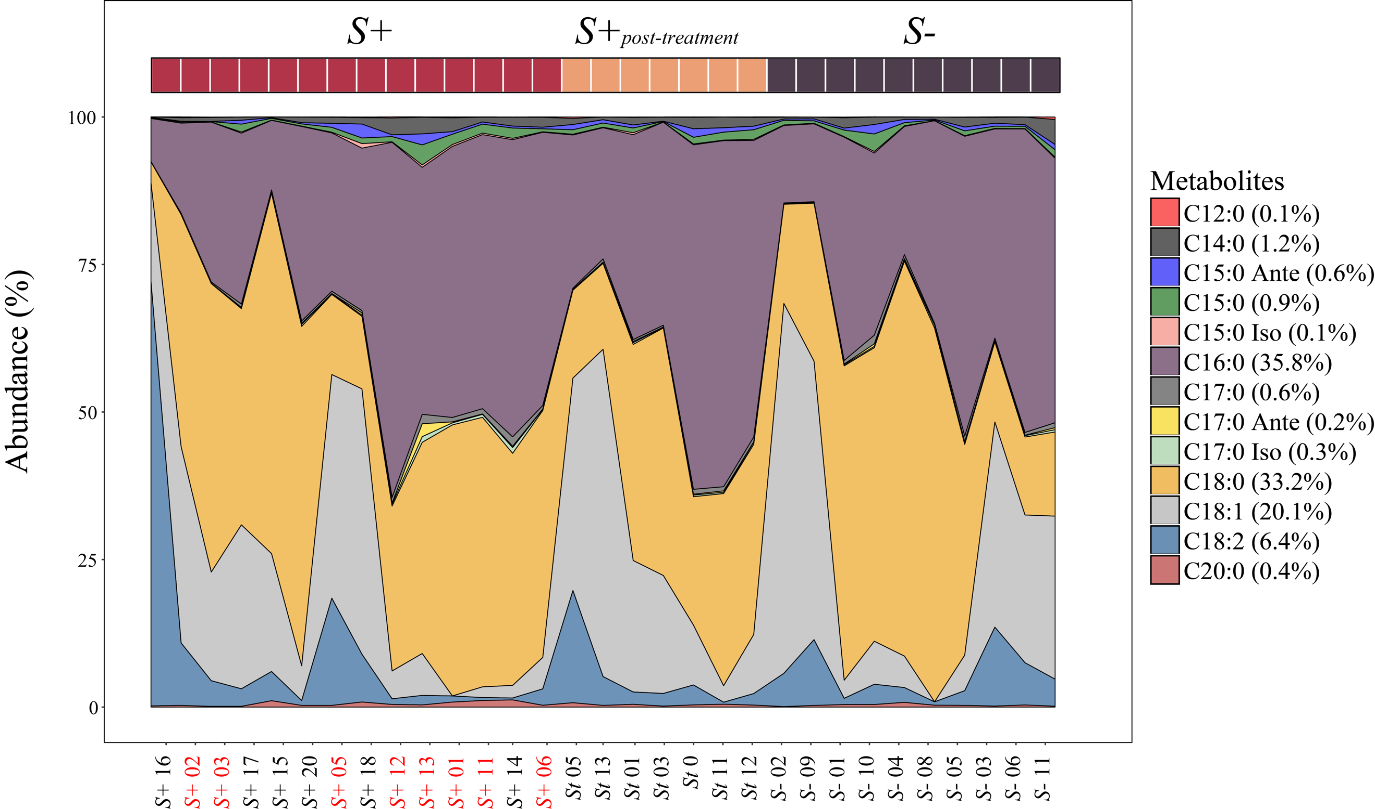
**
